# Postoperative Outcomes After Emergency Laparotomy in Nontrauma Settings: A Single-Center Experience

**DOI:** 10.7759/cureus.23426

**Published:** 2022-03-23

**Authors:** Awni D Shahait, Heather Dolman, Gamal Mostafa

**Affiliations:** 1 The Michael and Marian Ilitch Department of Surgery, Wayne State University School of Medicine, Detroit, USA

**Keywords:** preoperative evaluation, outcomes, mortality, non-trauma, emergency laparotomy

## Abstract

Introduction: Emergency laparotomy (EL) is a common operation that deals with a wide range of pathologies. Preoperative optimization is often lacking due to the urgent nature of the disease process with a reported mortality rate of up to 44%. This study examines the mortality of EL at an academic acute care surgery medical center.

Methods: A retrospective analysis of nontrauma EL from January 2008 to December 2013 was conducted. Data included demographics, clinical features, preoperative laboratory studies, comorbidities, time to surgery, ICU admission, and 30-day mortality.

Results: A total of 234 patients (123 males, 52.6%) were included in the study. EL was performed within four hours (immediate) of presentation in 93 (39.7%) patients, within 4-12 hours (early) in 53 (25.4%) patients, and within 12-24 hours (late) in 63 (30.1%) patients. Overall mortality was 16 (6.8%) at 30 days. Mortality was significantly higher with chronic obstructive pulmonary disease (p = 0.014), blood transfusion (p < 0.001), ICU admission (p < 0.001), ventilator days > four (p = 0.013), hyperlipidemia (p = 0.014), heart rate > 90 beats/minute (p = 0.003), temperature > 38°C or < 35°C (p = 0.013), and systolic blood pressure < 90 mmHg (p < 0.001).

Conclusion: EL can be performed with lower mortality than previously reported. Specific predictors of mortality are identified and can be used for risk assessment.

## Introduction

Emergency laparotomy (EL) performed for nontraumatic indications is one of the most common operations performed by acute care and general surgeons and represents about 11% of all surgical admissions, and more than 25% will require surgical intervention [1]. The procedure is often performed on older patients with many comorbidities [2].

Due to the emergent nature of the underlying pathologic process, the surgical team is often afforded insufficient time for preoperative physiologic optimization and forced to accept elevated perioperative risk when proceeding to the operating room. Consequently, prior studies have reported a mortality rate of 22-44% in patients over 65 years of age undergoing EL [1,2]. This study examines the rate and predictors of 30-day mortality of EL in an acute care surgery service at an urban academic medical center.

## Materials and methods

Study design

A retrospective review was conducted of patients undergoing nontrauma EL at the Detroit Receiving Hospital and the Harper University Hospital in Detroit, Michigan, from January 2008 to December 2013. Approval was obtained from Detroit Medical Center and Wayne State University institutional review boards (approval number: 090617M1E).

Inclusion and exclusion criteria

Adult patients, 18 years or older, who underwent exploratory laparotomy or emergent diagnostic laparoscopy converted to open within 24 hours of hospital admission or surgical consultation were included. Trauma and pediatric patients (<18 years of age) were excluded. In addition, patients undergoing gynecologic, transplant, vascular, and laparoscopic procedures were excluded.

Data collection

Patient demographics, including sex, age, race, BMI, and medical and social history, as well as immediate preoperative vital signs and laboratory values, were used to assess for preoperative risk. Operative and postoperative outcomes were reported, including time to surgery, intraoperative blood transfusions, ICU admission, prolonged ventilator support, and 30-day mortality.

Statistical analysis

Chi-square test was utilized to analyze categorical variables, while continuous variables were analyzed using unpaired Student’s t-test. Univariate analysis was conducted to further evaluate the 30-day mortality in relation to specific variables of interest. All analyses were performed using SPSS (IBM SPSS Statistics for Windows, version 23.0, IBM Corp., Armonk, NY). P < 0.05 was considered statistically significant.

## Results

A total of 234 patients met the selection criteria and were included in the analysis. Patient demographics, comorbidities, preoperative laboratory, and admission vital signs are listed in Table 1. The majority of the patients were African American (67.5%) and male (52.6%). The most common comorbidity was hypertension (51.7%).

**Table 1:** Patient characteristics and comorbidities comparing patients who died and the overall group. BMI: body mass index; IVDU: intravenous drug use; CAD: coronary artery disease; CHF: congestive heart failure; HTN: hypertension; COPD: chronic obstructive pulmonary disease; GFR: glomerular filtration rate; WBC: white blood cell count; HR: heart rate; RR: respiratory rate; SBP: systolic blood pressure.

Total number of patients = 234	All patients, N (%)	30-day mortality (%)	P-value
Age group			0.235
<70	196 (84.1)	12 (5.2)	
≥70	37 (15.9)	4 (1.7)	
Sex			0.258
Female	111 (47.4)	4 (1.7)	
Male	123 (52.6)	12 (5.1)	
Race			0.626
White	52 (22.2)	2 (0.9)	
African American	158 (67.5)	13 (5.6%)	
Hispanic	7 (3)	0 (0)	
Others	17 (7.3)	1 (0.4)	
BMI group			0.295
18.5-24.9	76 (32.5)	4 (1.7)	
25.0-29.9	82 (35)	5 (2.2)	
30.0-34.9	17 (7.3)	4 (1.7)	
≥35.0	24 (2.1)	2 (0.9)	
Current smoker	101 (43.2)	9 (3.9)	0.445
Current alcohol use	79 (33.8)	8 (3.4)	0.103
IVDU	51 (21.8)	6 (2.6)	0.043
Comorbidity			
Diabetes (insulin-dependent)	17 (7.3)	3 (1.3)	0.067
CAD	18 (7.7)	3 (1.3)	0.085
CHF	16 (6.8)	1 (0.4)	0.923
HTN	121 (51.7)	10 (4.3)	0.371
Hyperlipidemia	28 (12)	5 (2.1)	0.014
COPD	28 (12)	5 (2.1)	0.014
Cirrhosis	8 (3.4)	2 (0.9)	0.039
Renal failure (GFR < 15 mL/min/1.73 m^2^)	10 (6.9)	6 (2.6)	<0.001
Preoperative laboratory tests			
Albumin (g/dL)	3.1 (±0.8)	2.6 (±1.2)	0.102
WBC count > 12 or < 4 x 10^9^ per L	101 (43.2)	10 (4.3)	0.113
Hemoglobin (g/dL)	12.7 (±2.2)	11.8 (±2.7)	0.265
Preoperative vital signs			
HR > 90 beats/minute	90 (38.5)	12 (5.2)	0.002
RR > 20 breaths/minute	30 (12.8)	6 (2.6)	0.002
Temperature > 38°C or < 35°C	15 (6.4)	4 (1.7)	0.002
SBP < 90 mmHg	12 (5.1)	5 (2.2)	<0.001

Table 2 shows perioperative variables. The most common preoperative diagnosis was acute cholecystitis (36.3%), followed by perforated viscus (20.9%). Cholecystectomy (including open and converted cases) was the most commonly performed procedure (38.4%). Close to 40% of the patients went to the operating room within four hours of admission or surgical consultation, and over 60% within 12 hours. In terms of perioperative imaging workup, 38.8% of patients had abdomen and pelvis CT scan with IV contrast. Abdominal ultrasound was performed in 30.7% of cases, while plain abdominal radiograph was done in 25.2% of cases, with only one patient receiving no perioperative imaging.

**Table 2:** Perioperative variables comparing patients who died and the overall group.

Total number of patients = 234	N (%)	30-day mortality (%)	P-value
Preoperative diagnosis			0.550
Intestinal obstruction	28 (12)	1 (0.4)	
Acute cholecystitis	85 (36.3)	1 (0.4)	
Acute appendicitis	6 (2.6)	1 (0.4)	
Pneumoperitoneum	49 (20.9)	4 (1.4)	
Strangulated hernia	27 (11.5)	1 (0.4)	
Other	38 (16.2)	8 (3.4)	
Timing of surgery			0.100
Immediate (0-3.99 hours)	93 (39.7)	8 (3.8)	
Early (4-12 hours)	53 (22.6)	6 (2.9)	
Late (12-24 hours)	63 (26.9)	1 (0.5)	
Postoperative ICU admission	82 (35)	15 (6.4)	<0.001
Blood transfusion ≥ 2 units	45 (19.2)	11 (4.7)	<0.001

The overall 30-day mortality rate was 6.8%. Univariate analysis was conducted to further evaluate the 30-day mortality in relation to specific variables of interest as shown in Table 3. Glomerular filtration rate (GFR) < 15 mL/min/1.73 m^2^ (p < 0.001), chronic obstructive pulmonary disease (COPD) (p = 0.014), blood transfusion ≥ 2 units (p < 0.001), postoperative ICU admission (p < 0.001), ventilator support ≥ four days (p = 0.013), hyperlipidemia (p = 0.014), cirrhosis (p = 0.01), IV drug use (IVDU) (p = 0.047), preoperative heart rate (HR) > 90 beats/minute (p = 0.003), temperature > 38°C or < 35°C (p = 0.013), respiratory rate (RR) > 20 breaths/minute (p = 0.002), and systolic blood pressure (SBP) < 90 mmHg (p < 0.001) were all statistically significant. On the other hand, preoperative diagnosis and type of procedure had no effect on overall mortality.

**Table 3:** Summary of univariate analyses of perioperative variables on 30-day mortality. GFR: glomerular filtration rate; IVDU: intravenous drug use; COPD: chronic obstructive pulmonary disease; HR: heart rate; RR: respiratory rate; SBP: systolic blood pressure.

	P-value
Preoperative variables	
GFR < 15mL/min/1.73 m^2^	<0.001
COPD	0.014
Hyperlipidemia	0.014
IVDU	0.052
HR > 90 beats/minute	0.003
Temperature > 38°C or < 35°C	0.013
RR > 20 breaths/minute	0.002
SBP < 90 mmHg	<0.001
Operative and postoperative variables	
Transfusion ≥ 2 units	<0.001
ICU admission	<0.001
Ventilator ≥ 4 days	0.013

## Discussion

The acute care surgery model combines the provision of trauma care with emergency general surgery services. While much research has focused on improving outcomes and quality of surgical care in trauma, less attention has been afforded to such initiatives in general emergency surgery. Research has shown that both mortality and morbidity are substantially higher in emergency general surgery cases than elective procedures. This can at least in part be attributed to upregulation of the systemic inflammatory response in emergency surgical patients [3]. Additionally, this patient population harbors a higher comorbidity burden and lower baseline functional status [4].

In 2011, a large database study utilizing the American College of Surgeons National Surgical Quality Improvement Program (ACS NSQIP) demonstrated a 4.6 times higher mortality rate and 1.6 times more postoperative complications in general surgery patients undergoing emergent procedures as compared to nonemergent cases [5]. A large retrospective study examining surgical care within the Michigan Surgical Quality Collaborative found that while emergency procedures represented only 11% of total cases, they accounted for nearly half of mortalities and over a quarter of surgical complications [6]. Another ACS NSQIP-based analysis of 819 emergency general surgery cases from the Brigham and Women’s Hospital revealed 8.9% 30-day mortality, with a quarter of the patients having at least one postoperative complication, most commonly respiratory or wound issues [7]. Hence, it is paramount to characterize better demographic, clinical, and institutional factors that have predictive value in relation to morbidity and mortality in the emergency general surgery patient population and can perhaps be targeted in future quality improvement initiatives.

Our study focuses explicitly on EL, one of the most common emergency surgical procedures, as a way of studying factors related to mortality in the emergency general surgery population. A prospective observational study of 124 patients undergoing EL at a single UK hospital over five months found 16.8% 30-day mortality [2]. Data from 1708 EL patients from 35 UK National Health Service hospitals reported a 14.8% overall 30-day mortality rate [8]. A retrospective ACS NSQIP analysis from 2005 to 2009 involving 37,553 patients who underwent emergent laparotomy reported 14% mortality at 30 days [1]. In our study population, EL was associated with a mortality rate of 6.8% at 30 days. This is considerably lower than the rates previously cited in the literature. The reasons for such a stark difference are likely multifactorial. We believe that early identification of patients with acute abdomen, early surgical consultation, and prompt initiation of the surviving sepsis campaign protocol are the major contributing factors [9].

Furthermore, every effort was made to reduce the time from the initial patient presentation or surgical consultation to the operating room, with close to 40% of our patients undergoing operations within four hours. Despite not finding a statistically significant association between the timing of surgery and 30-day mortality in our study, we believe that this association exists based on prior research, and our study was not sufficiently powered to detect it [8]. We did notice a trend, though not statistically significant, with 53% of total deaths in patients undergoing surgery within the first four hours, 40% of deaths in the 4-12 hour surgical group, and 7% in the 12-24 hour group, which could be explained by having more time for proper resuscitation and initiation of antibiotics. Our study used different criteria for the classification of the timing of surgery compared to prior investigations, which utilized the UK National Confidential Enquiry into Patient Outcome and Death (NCEPOD) classification: immediate, urgent, and expedited [8]. In addition, unlike some prior studies, we included any cause of acute abdomen, including acute appendicitis and cholecystitis.

Based on our database analysis, predictors of 30-day mortality in our EL patient population were COPD, hyperlipidemia, low GFR, blood transfusion, ICU admission, prolonged mechanical ventilation, tachycardia, fever, or hypothermia, tachypnea, and hypotension. On the other hand, gender, age >75 years, ethnicity, BMI, preoperative diagnosis, American Society of Anesthesiologists (ASA) classification, presence of cardiovascular disease, smoking, or surgical site infection were not predictive of mortality at 30 days. We did not find a statistically significant association between advanced age and mortality in our analysis, which is in contrast to prior research and likely related to our small sample size. A 2003 study of 710 patients with acute abdomen did not find an association between increasing age beyond 70 years and mortality [10]. However, this study only included patients older than 70 years of age and did not compare mortality rates between elderly and non-elderly patients. An ACS NSQIP-based study from 2005 to 2008 reported that elderly patients (≥65 years of age) had a higher incidence of serious morbidity (OR: 1.17, p < 0.0001) and mortality (OR: 2.29, p < 0.0001) as compared to younger patients [11]. A small study from the UK looking at emergency general surgery outcomes in 85 patients found advanced age (≥70 years of age) to be an independent predictor of 30-day mortality (OR: 9.2, p = 0.004) [12].

Whereas we did not find a higher ASA class to predict 30-day mortality, this is in contrast to prior reported series [1,2,10,12-14]. We did, however, find multiple medical comorbidities including COPD and renal dysfunction be associated with increased mortality. Because patients with a higher comorbidity burden are usually assigned a higher ASA class, likely, our study was not sufficiently powered to detect a mortality difference when it comes to ASA classification. Of our patients, 36% were admitted to the ICU postoperatively, similar to previous studies. Like in prior research, ICU admission and prolonged mechanical ventilation were predictive of increased mortality [12]. The presence of sepsis was also a significant predictor of mortality, similar to reported data [1,8,12]. Length of stay was not a mortality predictor in our analysis, which is different from prior reports [15].

Limitations of our study include its observational retrospective design, small sample size, self-reported postoperative complications, as well as the lack of standardized protocol for EL at our institution.

## Conclusions

In this study, we showed that EL is associated with a high 30-day mortality rate. Certain perioperative factors that contribute to postoperative mortality were identified and can be used for individual patients' preoperative optimization and risk assessment.
